# Global genomic and molecular epidemiology of carbapenem-resistant *Enterobacter hormaechei*

**DOI:** 10.1038/s42003-026-09895-2

**Published:** 2026-04-15

**Authors:** Xiaoqian Long, Xiaohe Hu, Na Liu, Yujie Zhu, Guoping Zhao, Jiangang Ma, Biao Tang

**Affiliations:** 1 Key Laboratory of Systems Health Science of Zhejiang Province, School of Life Science, Hangzhou Institute for Advanced Study, University of Chinese Academy of Sciences, Hangzhou, Zhejiang China; 2 Xianghu Laboratory, Hangzhou, Zhejiang China; 3 Translational Medicine Research Center, Zhengzhou People’s Hospital, The Fifth Clinical College of Henan University of Chinese Medicine, Zhengzhou, Henan China; 4 Center for Supramolecular Chemistry & Catalysis and Department of Chemistry, College of Science, Shanghai University, Shanghai, China

**Keywords:** Bacterial genomics, Genome informatics

## Abstract

Carbapenem-resistant *Enterobacter hormaechei* (CR-Eh) represents a critical global health threat, especially when carrying mobile antimicrobial resistance genes (ARGs) like *bla*_NDM_ and *mcr*. This study characterized three clinical CR-Eh isolates in the context of global genomic datasets, identifying distinct patterns of resistance gene carriage. Notably, *bla*_NDM-1_ was found duplicated on the chromosome of strain BC18 but resided on plasmids in other isolates. In contrast, *mcr-9* was detected in a conserved genetic context on an IncHI2 plasmid. Analysis of publicly available genomes from 47 countries demonstrated that *bla*_NDM_ variants (primarily *bla*_NDM-1_ and *bla*_NDM-5_) and *mcr-9* are widely distributed globally. Within the available dataset, the isolates exhibited high genetic diversity, with numerous sequence types (including high-risk clones ST171 and ST190) and plasmids (IncX3/IncHI2) implicated in their spread. The strains carried an average of 14 ARGs each, and phylogenetic analysis suggested potential for cross-border and cross-habitat transmission. Collectively, our findings underscore that *E. hormaechei* is not merely a pathogen but a highly adaptable genetic reservoir for ARG accumulation and dissemination, highlighting the urgent need for systematic One Health genomic surveillance.

## Abstract (web summary)

Global genomic analysis of carbapenem-resistant *Enterobacter hormaechei* reveals *blaNDM-1* and *mcr-9* on IncHI2/IncX3 plasmids, with high-risk clones ST171/ST190 driving cross-border and cross-habitat transmission.

## Introduction

*Enterobacter hormaechei*, a key member of the clinically critical ESKAPE pathogen group and the *Enterobacter cloacae* complex^1^, represents a distinct and escalating threat as the third most prevalent nosocomial pathogen globally, after *Escherichia coli* and *Klebsiella pneumoniae*^2^. It causes bloodstream, abdominal, urinary tract, gastrointestinal, and pulmonary infections^3^. Due to its dual threat nature, *E. hormaechei* acts as an opportunistic pathogen in immunocompromised hosts, coupled with a clear tendency to develop a multidrug resistant (MDR) phenotype^4^. Carbapenems, once last-resort antibiotics for nosocomial infections, are increasingly compromised by the global spread of *bla*_NDM_ genes^5^. Surveillance studies have detected carbapenem-resistant *E. hormaechei* (CR-Eh) in multiple geographical regions (such as China^6^, France^7^, Brazil^8^), underscoring its potential status as a critical antimicrobial resistance (AMR) threat.

The plasmid-mediated spread of *mcr* genes further complicates treatment by reducing colistin efficacy, a cornerstone therapy for MDR Gram-negative infections^9^. Notably, co-occurrence of *bla*_NDM_ and *mcr* genes has been documented in *Klebsiella*^10^ and *Escherichia*^11^. The emergence and spread of strains are due to the high diversity of clonal lineages and carbapenemases. To date, 96 NDM variants (https://www.ncbi.nlm.nih.gov/pathogens/refgene/#gene_family:blaNDM) and 11 MCR variants (https://www.ncbi.nlm.nih.gov/pathogens/refgene/#MCR) have been identified worldwide, primarily disseminated via mobile genetic elements, especially IncX3, IncHI2, and IncFII-type plasmids^12–14^. Epidemiological investigations reveal that CR-Eh transmission is closely linked to high-risk clonal lineages (e.g., ST418^15^, ST78^16^, ST171^17^ and ST93^18^), which exhibit pandemic potential through plasmid-mediated horizontal gene transfer. Current research has been conducted on resistance mechanisms and epidemiological characterization of *E. coli*^19^, *K. pneumoniae*^20^ and *Salmonella*^21^. In contrast, *E. hormaechei* has not been systematically investigated, despite being defined by traits that predispose it to be an efficient resistance hub, including a broad ecological distribution^8^ and a propensity for stabilizing large plasmids^22^. This has resulted in a critical knowledge gap regarding the specific transmission mechanisms and evolutionary pathways of *bla*_NDM_ and *mcr* genes within this species.

In this study, we combined detailed genomic analysis of three clinical CR-Eh isolates with a global bioinformatic investigation to elucidate the mechanisms underpinning the success of *E. hormaechei* as a vehicle for high-threat resistance genes. We explicitly acknowledge that the NCBI dataset represents a convenience sample with certain geographical and source biases, and our interpretations are framed within this limitation. Our analysis focused on the following scientific objectives: (1) molecular genetic characterization of *bla*_NDM_ and *mcr* resistance genes and their dissemination characteristics; (2) spatiotemporal distribution of dominant clones and plasmid types; and (3) phylogeny of resistant strains. The research results will provide a molecular epidemiological basis for clinical treatment and inform precision control strategies for AMR bacteria, with a specific focus on elucidating the mechanisms behind the success of *E. hormaechei* as a vehicle for these high-threat resistance genes.

## Results

### Isolation of strains and antimicrobial resistance

Three CR-Eh strains—BC18, BC30, and BC37—were resistant to ampicillin, amoxicillin-clavulanic acid, gentamicin, sulfisoxazole, trimethoprim/sulfamethoxazole, ceftiofur, ceftazidime, enrofloxacin, ofloxacin, and meropenem. In addition, isolates BC18 and BC37 were resistant to tetracycline, florfenicol, and spectinomycin. Notably, isolates BC30 and BC37 remained susceptible to colistin with a minimum inhibitory concentration (MIC) of 1 μg/mL, whereas BC18 exhibited an elevated MIC of 4 μg/mL, reaching the clinical breakpoint for resistance (Supplementary Data 1).

### Genomic characterization of isolates

All three clinical isolates carried the *bla*_NDM-1_ gene, with strain BC18 also carrying the *mcr-9* gene. The *mcr-9* and *bla*_NDM-1_ genes are respectively located on the plasmid pBC18-494k and the chromosome of strain BC18 (Supplementary Data 2). S1 nuclease pulsed-field gel electrophoresis (S1-PFGE) and Southern blot indicated that the *bla*_NDM-1_ gene in BC30 is located on a plasmid (Supplementary Fig. 1). *bla*_NDM-1_ of strain BC37 was located on the plasmid pBC37-81k (Supplementary Data 2). Besides *bla*_NDM-1_ and *mcr-9* genes, 17 ARGs from 7 classes were identified in the three isolates. Among them, aminoglycoside and folate pathway antagonist resistance genes were the most common. Multilocus sequence type (MLST) analysis assigned ST231, ST78 and ST171, with ST78 and ST171 representing internationally prevalent clones (Supplementary Data 2), and all of them carried the *nlpI* virulence gene, which can affect cell wall metabolism via MepS and PBP4 endopeptidases.

### Plasmid comparison and genetic environment analysis of *bla*_NDM-1_ gene

**Fig. 1:**
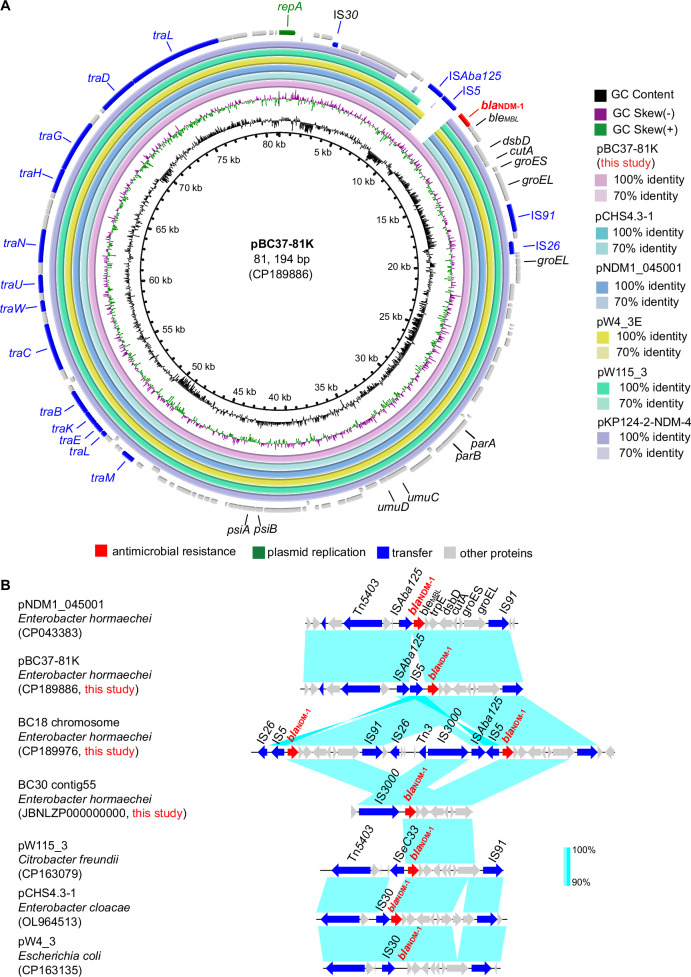
Genetic characteristics of *bla*_NDM-1_ gene. **A** Circular comparison of pBC37-81K with similar plasmids, pCHS4.3-1, pNDM1_045001, pW4_3E, pW115_3 and pKP124-2-NDM-4. **B** Linear comparison of pNDM1_045001, pBC37-81k, BC18 chromosome, BC30 contig55, pW115_3, pCHS4.3-1 and pW4_3E. Blue shaded areas indicate > 90% DNA identity between sequences. The red arrow indicates the ARGs, the blue arrow indicates the mobile elements, the green arrow indicates the plasmid replication, and the grey arrow indicates other proteins.

Comparative plasmid analysis revealed that strain BC37 carries the *bla*_NDM-1_ gene on an untypable plasmid, designated pBC37-81k (81,194 bp). The plasmid shares high plasmid backbone (95%-99% coverage, 99.9%-100% homology) with several clinical  relevant *Enterobacteriaceae* plasmids, including *E. cloacae* pCHS4.3-1, *E. hormaechei* pNDM1_045001, *E. coli* pW4_3, *Citrobacter freundii* pW115_3, and *K. pneumoniae* pKP124-3-NDM-4, suggesting extensivehorizontal dissemination of *bla*_NDM-1_ among diverse strains. These plasmids share replication initiator *repA*, conjugative transfer elements and *psiA/B* element (Fig. 1A), and the main difference lies only in the insertion element upstream of the *bla*_NDM-1_ gene.

The genetic environment of the *bla*_NDM-1_ gene was similar in BC37 and BC18: IS*5*–*bla*_NDM-1_–*ble*_MBL_–*trpE*–*dsbD*–*cutA–groES–groEL*–IS*91*. In BC37, *bla*_NDM-1_ is plasmid-borne, whereas in strain BC18, it is integrated into the chromosome in two copies. BC30 differs from strain BC18 in that on BC30, two insertion elements are missing between IS*3000* and *bl*a_NDM-1_ (Fig. 1B). Conjugation frequencies were (3.23 ± 0.02) × 10^-5^ for strain BC30 and (4.60 ± 0.06) × 10^−5^ for strain BC37.

### Plasmid comparison and genetic environment analysis of the *mcr-9* gene

The *mcr-9* gene was located on a IncHI2-type plasmid, pBC18-494k (494, 411 bp), carrying IncHI2 and IncHI2A replicons (Supplementary Data 2). Its location was confirmed by S1-PFGE and Southern blot (Supplementary Fig. 1). Conjugation frequency was (5.67 ± 0.29) × 10^−5^. In addition to the *mcr-9* resistance gene, many other ARGs were found on pIncHI2-mcr-9 (Supplementary Data 2). Meanwhile, it was found that pIncHI2-mcr-9 contained multiple IS*26*, IS*903* and IS*5075* insertion elements.

**Fig. 2:**
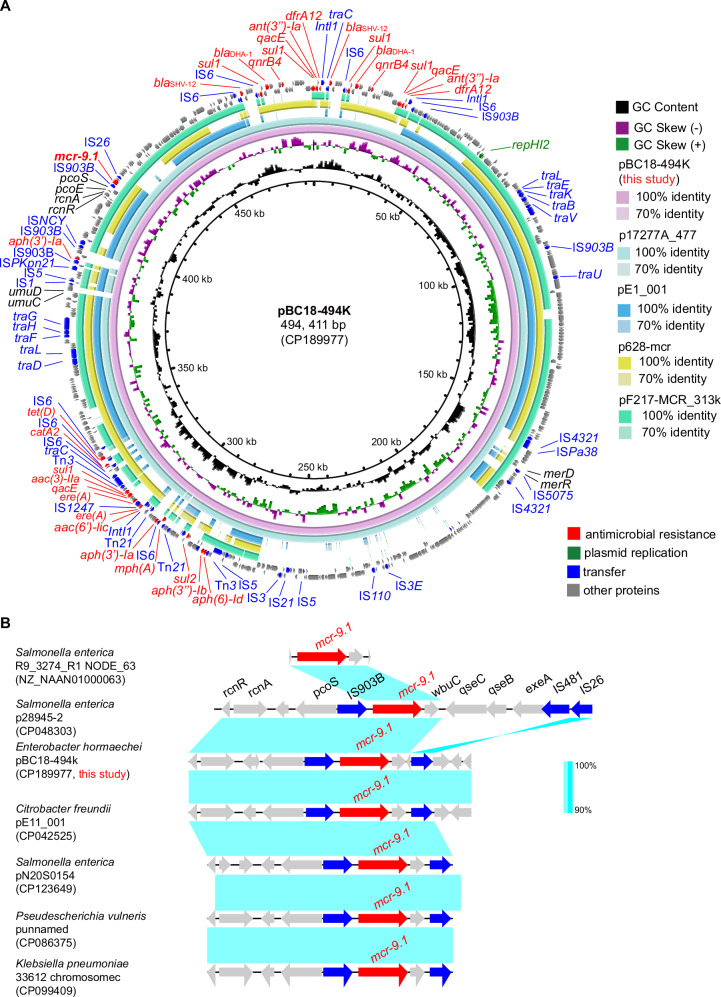
Genetic features of *mcr-9* gene. **A** Circular comparison of pBC18-494k with similar plasmids, p17277A_477, pE1_001, p628-mcr and pF217-MCR_313k. **B** Linear comparison of R9_3274_R1 NODE_63, p28945-2, pBC18-494k, pE1_001, pN20S0154, punnamed, and 33612 chromosome. Blue shaded areas indicate >90% DNA identity between sequences. The red arrow indicates the ARGs, the blue arrow indicates the mobile elements, the green arrow indicates the plasmid replication, and the grey arrow indicates other proteins.

Comparative genomic analysis showed similarity to other plasmids, with a distinct region shared with p17277A_477. Downstream of *mcr-9* was an IS*26*-bounded resistance gene cluster including a class I integron. Moreover, besides the *mcr-9* genetic context, these plasmids shared a conserved IncHI2 backbone with a complete conjugative system and heavy metal resistance determinants (Fig. 2A). The *mcr-9* gene is widely circulated in other species in the *rcnR-rcnA-hp-pcoS-*IS*903B-mcr-9-wbuC-*IS*26* structure. Notably, the *Salmonella* p28945-2 plasmid contains *qseB/C* and *exeA* genes inserted between *wbuC* and IS*26*, these elements are absent in pBC18-494k (Fig. 2B). The co-localization of *mcr-9* with numerous other resistance genes on a single conjugative plasmid suggests a powerful framework for co-selection under antimicrobial and heavy metal pressures, which may underpin the persistence and spread of this resistance determinant in *E. hormaechei* populations.

### Global distribution and variant characteristics of *bla*_NDM_- and *mcr*- harboring *E. hormaechei*

**Fig. 3:**
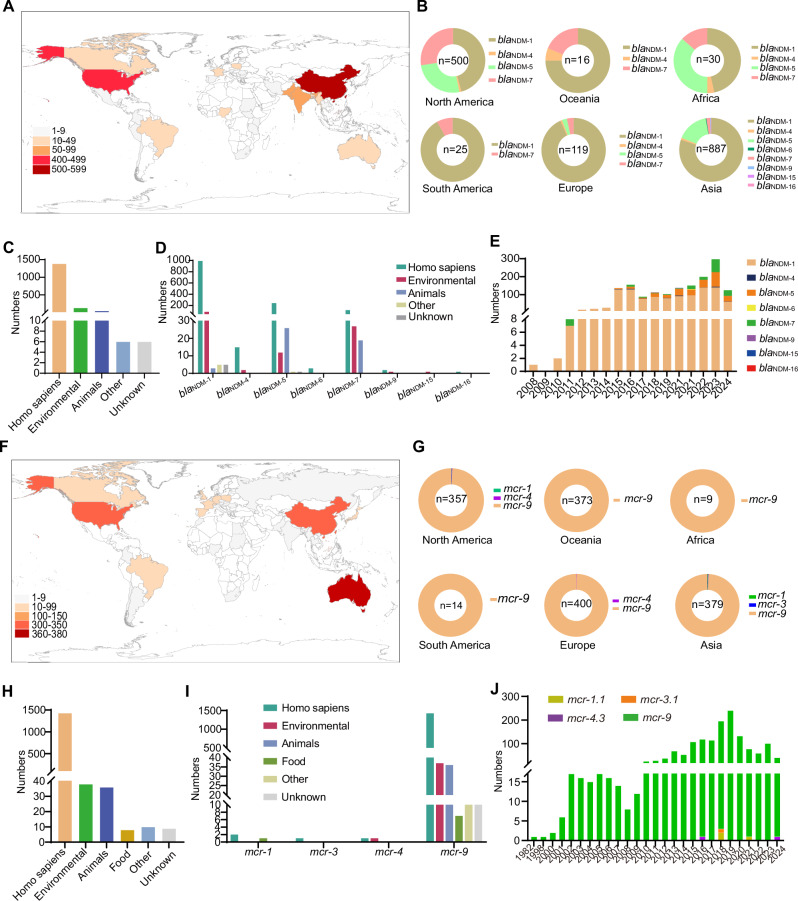
Global distribution of strains of *E. hormaechei* carrying *bla*_NDM_ and *mcr* genes. **A** Global distribution of 1577 strains of *E. hormaechei* carrying *bla*_NDM_ gene. The redder the color, the greater the number of positive strains reported in the corresponding country. **B** Each hollow pie chart shows the distribution of the *bla*_NDM_ variant on different continents. **C** The histogram shows the origin distribution of 1577 strains carrying *bla*_NDM_ gene. The *X* axis indicates the source and the *Y* axis indicates the number of positive strains. **D** The bar chart shows the origin of different *bla*_NDM_ variants, with the *X* axis representing the origin and the *Y* axis representing the number of *bla*_NDM_ gene carrying isolates. **E** The bar chart shows the number of isolates of *bla*_NDM_-carrying *E. hormaechei* worldwide in different years. **F** Global distribution of 1532 strains of *E. hormaechei* carrying *mcr* gene. **G** Each hollow pie chart shows the distribution of *mcr* variants across continents. **H** The bar chart shows the origin distribution of 1532 strains carrying *mcr* gene. **I** The bar chart shows the origin of the different *mcr* variants. **J** The bar chart shows the global number of *mcr*-carrying *E. hormaechei* isolates in different years.

To clarify the global prevalence trends of *bla*_NDM_ and *mcr* genes in *E. hormaechei*, we conducted a comprehensive investigation on this species. Our dataset comprised 1577 *bla*_NDM_-harboring and 1532 *mcr*-harboring *E. hormaechei* isolates, which revealed several noteworthy patterns. It is important to note that these distributions may be influenced by regional disparities in sequencing efforts. *bla*_NDM_-harboring strains were predominantly reported in Asia (56.25%, with the majority from China) and North America (31.71%), suggesting potential intercontinental transmission risks (Fig. 3A–D). Since their initial report in India in 2008, these strains have been reported globally within 15 years, with a peak in reporting frequency in 2023 (Fig. 3E). In contrast, *mcr*-harboring strains showed a more balanced distribution across Europe (26.11%), Asia (24.74%), Oceania (24.35%), and North America (23.30%), with Australia being a major source of reports (24.35%). Despite an early detection in a Canadian food sample (1982), the vast majority (>90%) of subsequent reports were from clinical settings (Fig. 3F–I).

Genetic diversity was markedly greater among *bla*_NDM_ variants, with eight subtypes including dominant *bla*_NDM-1_ (69.75%), *bla*_NDM-5_ (18.07%), and *bla*_NDM-7_ (10.59%) showing geographic specificity (e.g., *bla*_NDM-6_/*bla*_NDM-9_ exclusive to China) and accelerating emergence timelines (*bla*_NDM-7_ was reported merely three years after the first report of *bla*_NDM-1_) (Fig. 3E). *mcr-9* predominated (99.67%) since 1982, with sporadic variants (*mcr-1/mcr-3/mcr-4*) emerging post-2016 (Fig. 3J). Critical transmission differences emerged: *bla*_NDM_ variants spread through environmental (2013) and animal (2015) reservoirs before clinical establishment, while *mcr* variants remained clinically focused (Fig. 3E, J). These patterns identify Asia as the epicenter of both *bla*_NDM_ diversity and rapid transmission, while highlighting *mcr-9* gene’s exceptional stability and the growing threat from accelerating resistance gene evolution.

### Sequence type analysis of *bla*_NDM_- and *mcr*-harboring *E. hormaechei*

**Fig. 4:**
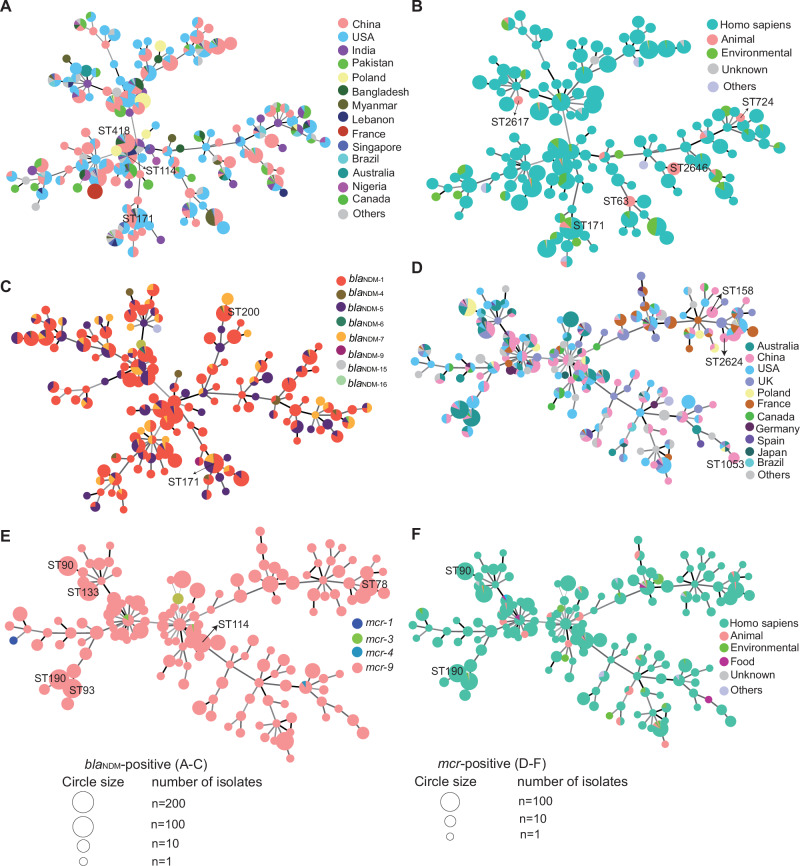
Minimum spanning tree of *bla*_NDM_- and *mcr*- producing *E. hormaechei* isolates based on MLST. **A** STs distribution of *bla*_NDM_-producing isolates in various countries. Different colors represent different countries. **B** STs distribution of *bla*_NDM_-producing isolates in various hosts. Different colors represent different hosts. **C** STs distribution of *bla*_NDM_-producing isolates in different *bla*_NDM_ variants. Different colors represent different *bla*_NDM_ variants. **D** STs distribution of *mcr*-producing isolates in various countries. Different colors represent different countries. **E** STs distribution of *mcr*-producing isolates in different *mcr* variants. Different colors represent different *mcr* variants. **F** STs distribution of *mcr*-producing isolates in various hosts. Different colors represent different hosts. Nodes represent STs, with node size proportional to the number of isolates within each ST. Sample sizes: *n* = 1503 for *bla*_NDM-_positive isolates with identified ST types (**A**–**C**); *n* = 1471 for *mcr*-positive isolates with known ST types (**D**–**F**).

Among 1577 *bla*_NDM_-harboring strains, 95.3% (*n* = 1503) were typed, identifying 148 STs. The ST171 clone was identified in genomes from 12 countries across 5 continents, with sources encompassing humans, animals, and the environment, demonstrating its broad geographical dissemination and multi-host adaptability (Fig. 4A–C). Secondary prevalent clones ST418 (6.28%) and ST114 (5.26%) showed distinct geographic distributions, with ST418 prominent in China where it accounted for 11.83% of isolates. Among the available data, the United States and China showed the highest ST diversity among *bla*_NDM_-harboring isolates (Fig. 4A), correlated with extensive sequencing efforts. While ST171 dominated across sources, several STs (ST2617, ST63, ST2646, and ST724) have thus far only been detected in animal samples (Fig. 4B). ST171 predominated in *bla*_NDM-1_, *bla*_NDM-5_, and *bla*_NDM-7_ carriers, while ST200 dominated in *bla*_NDM-4_ strains (Fig. 4C). Based on the available data, an association was observed between *bla*_NDM-1_ and three frequently reported STs (ST171, ST114, and ST418), with plasmid profiling revealing IncX3 as the predominant plasmid in ST171 and ST114 strains, while IncFIB (pHCM2) was most common in ST418 and ST78 isolates (Supplementary Data 3).

*mcr-*harboring strains exhibited high ST diversity (1471 strains, 146 STs), with lineages (ST190, ST90, ST93, ST78, ST114, ST133) strongly linked to *mcr-9* and IncHI2/2A plasmids (Supplementary Data 3). The U.S. had the highest ST diversity (67 STs, 45.89% of global diversity), followed by China (66 STs, 45.21%) and the UK (46 STs, 31.51%) (Fig. 4D). Some STs (such as ST158, ST2624, and ST1053) have thus far only been reported in China (Fig. 4D). Notably, *mcr-9* appeared in 146 STs, indicating broad host adaptability (Fig. 4E). Within the available dataset, ST190 and ST90 have primarily been reported from humans in Australia. This observed pattern, though potentially influenced by regional surveillance efforts, may suggest localized expansion of these clones, warranting further investigation (Fig. 4F). Specifically, ST65, the first known ST, diversified during transmission but was gradually supplanted by emerging types that displayed regional outbreak patterns (Supplementary Data 3).

### Correlations between ARGs, plasmid replicons, and virulence genes

**Fig. 5:**
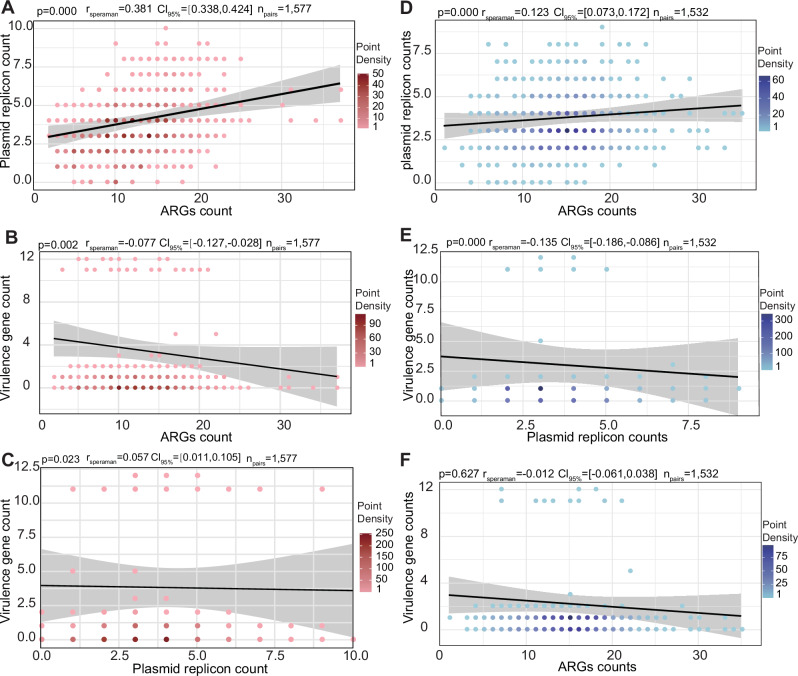
Scatter plot and correlation analysis between antimicrobial resistance genes, plasmid replicons, and virulence factors. **A** The correlation between plasmid replicons and ARGs counts of strains carrying the *bla*_NDM_ gene. **B** The correlation between ARGs and VFs counts of strains carrying the *bla*_NDM_ gene. **C** The correlation between plasmid replicons and VFs counts of strains carrying the *bla*_NDM_ gene. **D** The correlation between plasmid replicons and ARGs counts of strains carrying the *mcr* gene. **E** The correlation between plasmid replicons and VFs counts of strains carrying the *mcr* gene. **F** The correlation between ARGs and VFs counts of strains carrying the *mcr* gene. CI_95%_, 95% confidence interval. Correlations were assessed using Spearman’s rank correlation test. Sample sizes: *n* = 1577 for *bla*_NDM-_positive isolates (**A**–**C**); *n* = 1532 for *mcr*-positive isolates (**D**–**F**). Shading represents the 95% confidence interval.

We analyzed the associations between ARGs, plasmid replicons, and virulence factors (VFs) in global isolates. In *bla*_NDM_-harboring isolates (*n* = 1577), a significant positive correlation was observed between the number of ARGs and the number of plasmid replicons (r = 0.381, *p* < 0.001) (Fig. 5A). However, analysis revealed an inverse relationship between virulence factors and ARGs (Fig. 5B). In comparison, the virulence factors showed no clear association with plasmid replicons (Fig. 5C). Notably, 3.7% (58/1577) of *bla*_NDM_-positive isolates carried the *bla*_NDM_ gene on a plasmid, with the majority (81%) being the *bla*_NDM-1_ subtype. This subtype exhibited strong plasmid associations, primarily with IncX3 (42.6% of *bla*_NDM-1_ plasmids), IncHI2/2A (8.5%), and IncN2 (10.6%) replicon types. Striking plasmid subtype specificity was observed: *bla*_NDM-5_ was exclusively associated with IncX3 plasmids, while *bla*_NDM-4_ was solely linked to IncA/C2 plasmids in our dataset (Supplementary Data 4).

In *mcr*-positive isolates (*n* = 1532), a weak positive correlation was observed between ARGs and plasmid replicons (r = 0.123, *p* < 0.001), whereas plasmid replicons showed a negative correlation with VFs (r = 0.135, *p* < 0.001) (Fig. 5D, E). Furthermore, no significant correlation was detected between ARGs and VFs (r = 0.012, *p* > 0.05) (Fig. 5F), suggesting that pathogenicity and AMR may disseminate independently. In *mcr-9*-positive strains, we identified 98 plasmids from 98 isolates that carried defined plasmid replicon types, with 93.9% (92/98) harboring the IncHI2-IncHI2A plasmid replicon combination (Supplementary Fig. 2A). These plasmids carried an average of 11.5 ARGs (Supplementary Data 5), encompassing 63 distinct ARG types. The sulfonamide resistance gene *sul1* was the most prevalent (*n* = 186) (Supplementary Fig. 2B). In addition to its strong association with *mcr-9*, the IncHI2-IncHI2A replicon showed high-frequency co-occurrence with *sul1* (91.3%, 84/92), *aph(3”)-Ib* (58.7%, 54/92), and *aph(6)-Id* (58.7%, 54/92) (Supplementary Fig. 2C, Supplementary Data 6). Specifically, the IncHI2-IncHI2A replicon was also frequently linked to ESBL and other *β*-lactamase genes, including *bla*_SHV-12_ (48.9%, 45/92), *bla*_TEM-1B_ (43.5%, 40/92), *bla*_OXA-1_ (10.9%, 10/92), and *bla*_CTX-M-9_ (8.7%, 8/92) (Supplementary Fig. 2C, Supplementary Data 6).

### Phylogenetic analysis of ST

**Fig. 6:**
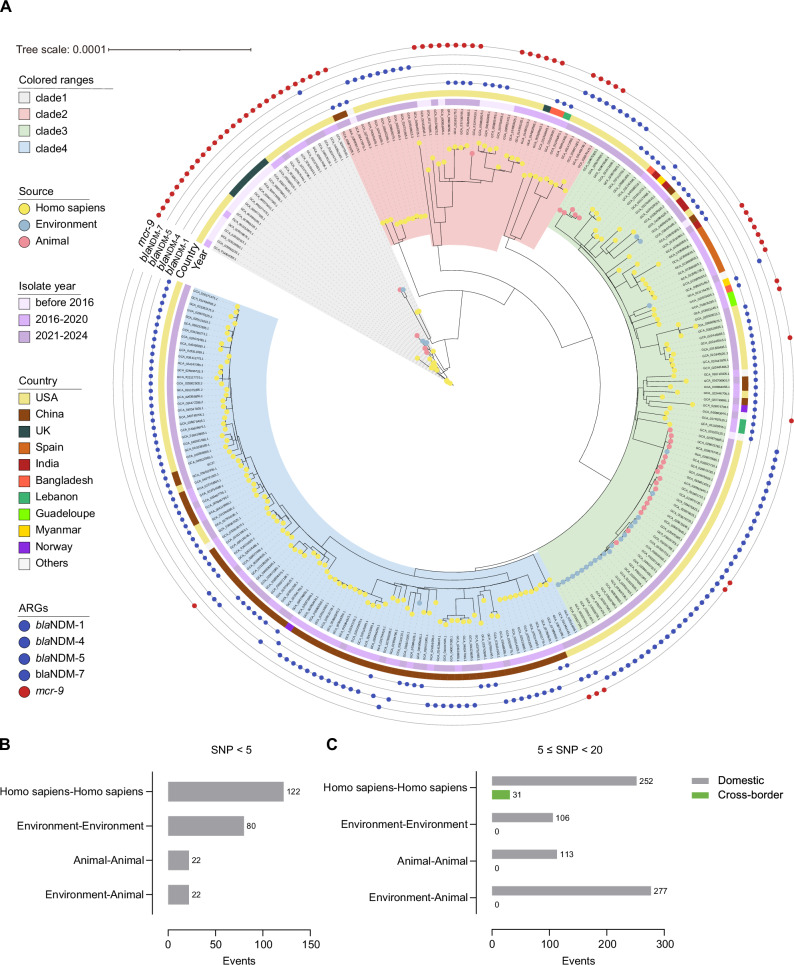
The transmission dynamic characteristics of *E. hormaechei* ST171. **A** Phylogenetic tree of 250 isolates belonging to ST171. **B** Transmission events with SNP distances <5. **C** Transmission events between 5 ≤ SNP < 20. Different branch colors represent different clade, and the circles of different colors on the branches represent different source. The phylogenetic tree, from the inner to the outer circle, successively contains the accession number of the strain, the year of strain isolation, the country of strain isolation, *bla*_NDM-1_, *bla*_NDM-4_, *bla*_NDM-5_, and the carrying status of the *mcr-9* gene.

We analyzed transmission dynamics and evolution in three distinct STs (ST171/ST78/ST231, SNPs>1000). Bayesian analysis revealed that ST171 diverged into four clades: clade1 and clade3 exhibited cross-habitat transmission (human-animal-environment), while clade2 and clade4 were almost entirely derived from human. Evolutionary tracing suggested ST171 originated in Europe (early UK strains), with clade1-3 concentrated in the U.S. and clade4 driving China’s epidemic (Fig. 6A). The analysis of the transmission network showed that under the strict threshold (SNPs < 5), there were 22 animal-environmental transmission incidents (Fig. 6B, Supplementary Data 7). When the threshold was relaxed to 20 SNPs, the 277 cases of cross-habitat transmission mainly followed the environmental to animal route. In human-to-human close contact transmission, intra-regional transmission was dominant (283 cases), but cross-border transmission cases emerged (the SNP difference between human samples from China and the United States was the smallest, SNPs = 9) (Fig. 6C, Supplementary Data 8). Significantly, in 2020, the clinical strain BC37 was involved in 1 case of local transmission and 3 cases of cross-border transmission (Supplementary Data 8). Importantly, all cross-border transmissions of ST171 are achieved through human-to-human transmission, and the ARGs show an evolutionary gradient: gradually superimposed from the single *mcr-9* of clade1 to the full-spectrum *bla*_NDM_ subtype of clade4 (Fig. 6A, C).

Similarly, ST78 strains formed three distinct branches, primarily comprising human clinical isolates (95.2%). However, both animal (France, 2015) and environmental (China, 2022) isolates were present simultaneously in clade1. These non-clinical isolates were clustered in the same evolutionary branch as the clinical isolates, confirming that this lineage has the ability to spread across species. The isolates of American origin located in clade2 showed accelerated evolution, while the strains of origin from southern France (2016–2020) located in clade3 showed spatiotemporal clustering linked to rising *mcr-9* prevalence in clinical (Supplementary Fig. 3A). For ST231, two major branches were identified, with human clinical isolates predominating (82.9%). Environmental strains peaked in 2016-2017, highlighting environmental transmission risks. Asia emerged as the primary endemic region, with Indian strains displaying high genetic diversity-harboring *bla*_NDM-1_, *bla*_NDM-4_, and *bla*_NDM-5_, correlating with regional carbapenem use. A unique Canadian environmental strain carried *bla*_NDM-7_, while clade2 analysis traced 15 human and environmental strains to a common 2015 ancestor, confirming trans-host transmission (Supplementary Fig. 3B).

## Discussion

The convergence of carbapenem and colistin resistance mechanisms in Gram-negative pathogens represents a critical threat to global health. Our study, integrating genomic analysis of clinical isolates with a global dataset, confirms that *E. hormaechei* is not merely another vehicle for these resistance genes but constitutes a highly adaptable and proficient genetic reservoir for their accumulation and dissemination. The high prevalence of *bla*_NDM_ variants and near dominance of *mcr-9* in this species, as observed in our data, prompts a crucial question: what distinct evolutionary and ecological pressures have favored *E. hormaechei* to emerge as such a concerning reservoir for these high-risk resistance genes?

The dissemination of *bla*_NDM-1_, once confined to clinical settings, now traverses a complex hospital-community-environmental network, as evidenced by its detection in diverse niches. This expansion underscores the role of *E. hormaechei* as a bridge species, facilitating the flow of resistance across One Health compartments. While *bla*_NDM-1_ is often associated with well-defined plasmid types like IncFII or IncL/M in other *Enterobacteriaceae*^19^, its presence in our strain BC37 on a novel, untypable plasmid is noteworthy. The conjugation frequency we observed (~10^−5^) is consistent with many successful conjugative plasmids^23^, suggesting this unclassified vector is functionally competent. The growing number of similar untyped plasmids harboring *bla*_NDM_ in databases suggests that *E. hormaechei* may utilize diverse and flexible genetic strategies for resistance gene acquisition, possibly leveraging its genomic plasticity to stabilize and maintain these exogenous elements^24,25^. Beyond this genetic flexibility, our data reveal broader evolutionary trends: while *bla*_NDM-1_ remains dominant, the alarming growth rates of *bla*_NDM-5_ and *bla*_NDM-7_ indicate active adaptation. This is further evidenced by a high degree of gene-plasmid compatibility, such as the strong association between *bla*_NDM_ variants and the compact, highly efficient IncX3 plasmids.

Our findings, consistent with recent studies, demonstrate a distinct preference of the *mcr-9* gene for *E. hormaechei*, likely attributable to its unique genetic background and host adaptation advantages. Furthermore, the expression of *mcr-9* is tightly regulated by systems such as *qseC/B*, remaining silent under non-selective conditions to minimize fitness costs to the host^26^. Although this gene was first identified in *Salmonella*, current evidence suggests it originates from *Buttiauxella* spp^26^; the phylogenetic proximity of this genus to *Enterobacter spp*. explains its prevalence in the latter, and its codon usage preference is conducive to the successful colonization of *mcr-9* in *Enterobacter* species hosts^27^.

In contrast, its expression may be dysregulated or toxic in other bacterial backgrounds, reducing fitness^28^. The genetic context of *mcr-9* is also relatively conserved, further enhancing its stability^29^. The global spread of these genes is further amplified by their association with specific, efficient plasmid vectors. *mcr-9* is predominantly linked to large IncHI2 plasmids, which frequently serve as MDR reservoirs^29,30^. In our collection, the dominant IncHI2-IncHI2A replicon was found to co-harbor a wide array of resistance genes alongside *mcr-9*, most notably the sulfonamide resistance gene *sul1* (91.3%), the aminoglycoside resistance genes *aph(3”)-Ib* and *aph(6)-Id* (each 58.7%), and clinically relevant ESBL genes such as *bla*_SHV-12_ (48.9%) and bla_TEM-1B_ (43.5%). Approximately 83% of *mcr-9*-carrying IncHI2 plasmids harbor one to five additional ESBL or carbapenemase genes^29^, and this phenomenon is generally consistent with our research. However, there are also exceptional cases where no other ARGs were found on IncHI2 or other plasmids (such as IncFIB) carrying the *mcr-9* gene^27,29^. More importantly, the IncHI2 plasmid almost always co-carries heavy metal resistance genes, enabling the strain to survive and spread even without antibiotic pressure^22,31^. Although large plasmids often exhibit lower conjugation frequencies, the previously reported conjugation rates (10^−5^ to 10^−6^) are highly effective for plasmids of this size^32^, attributable to a robust type IV secretion system and complex maintenance modules encoded by the conserved IncHI2 backbone^29^.

It is worth noting that the prominent reporting of strains with *bla*_NDM_ or *mcr* genes in the U.S., along with the high ST diversity observed there and in China, is largely driven by these countries’ significant investments in genome sequencing. Clonal expansion acts as a second powerful engine driving CR-Eh dissemination. The success of ST171 is not merely due to human activity but is underpinned by its remarkable genomic plasticity and broad ecological tolerance^17^. This enables it to serve as an efficient vector, efficiently obtaining and stably maintaining a rich pool of ARGs. Phylogenetic analysis reveals closely related strains isolated from humans, animals, and the environment, highlighting a potential risk of cross-compartment transmission. We observed an evolutionary gradient in ST171, characterized by the progressive accumulation of resistance determinants, which culminated in Chinese clade4 strains possessing full-spectrum resistance. This suggests a progressive adaptation within this lineage, potentially in response to intense antimicrobial selective pressures. Similarly, ST78, though less prevalent in our study, is a known vector for MDR plasmids^17^. The emergence of ST231, co-harboring *bla*_NDM-1_ and *mcr-9* with high conjugation efficiency, signals the ongoing evolution of new threatening clones. The synergy between high-risk plasmids and high-risk clones creates opportunities for the rapid and sustained global spread of AMR. While our study elucidates key mechanisms behind the success of CR-Eh, several questions remain. Future work should experimentally determine the fitness cost of these large MDR plasmids in *E. hormaechei* compared to other *Enterobacteriaceae*, to quantify its competitive advantage as a reservoir.

In conclusion, our integrated analysis of clinical isolates together with publicly available genomic datasets reveals the key mechanisms and vectors (notably IncHI2, IncX3 plasmids) that facilitate the dissemination of CR-Eh. We further document the potential co-transfer of *bla*_NDM-1_ and *mcr-9*, and identify high-risk clonal lineages (e.g., ST171 and ST190) with clear potential for cross-border and cross-habitat transmission. At the same time, our work also highlights the substantial sampling and reporting biases embedded within current genomic databases. These findings underscore the urgent need for establishing a systematic, One Health-based genomic surveillance system to more accurately monitor and mitigate the dissemination of CR-Eh.

## Methods

### Collection and isolation of strains

Three CR-Eh clinical isolates were collected between August and October 2021 from a tertiary hospital in Henan Province, China. BC18 was isolated from a urine sample of a 72-year-old male postoperative patient with a bladder tumor with hematuria and a urinary stent, clinically diagnosed with a urinary tract infection. BC30 came from a sputum sample of a 79-year-old female with pulmonary infection and coronary artery disease. BC37 was isolated from a blood sample of a 47-year-old male patient with pulmonary infection. All strains were identified as *E. hormaechei* by MALDI-TOF MS and confirmed via whole-genome sequencing. Biological samples were collected for bacterial isolation following comprehensive verbal informed consent. During this process, participants were fully briefed on the study’s scope, the voluntary nature of their involvement, and the use of their de-identified leftover clinical specimens. This procedure was conducted in accordance with medical ethics guidelines and approved by the Research Ethics Committee of Zhengzhou People’s Hospital (KY2022-013-01).

### Antimicrobial susceptibility test

MICs of 14 antibiotics were determined using the microbroth dilution method, following CLSI M100-ED32 guidelines^33^. Antibiotics included: ampicillin, amoxicillin/clavulanic acid, gentamicin, spectinomycin, tetracycline, florfenicol, sulfisoxazole, trimethoprim/sulfamethoxazole, ceftiofur, ceftazidime, enrofloxacin, ofloxacin, meropenem, and colistin. *E. coli* strain ATCC 25922 was used as quality control (Supplementary Data 1).

### Conjugation transfer assay

The conjugation transfer assay was conducted using the filter membrane method to evaluate the transferability of plasmids^34^. Conjugation experiments were performed using the rifampicin-resistant *E. coli* C600 as the recipient strain and isolates BC18, BC30, and BC37 as the donors. The ratio of donor: recipient = 1:3. Transconjugants screening was conducted using resistant plates containing sodium azide (MIC = 100 mg/L), sodium azide (MIC = 100 mg/L) and meropenem (MIC = 2 mg/L) or colistin (MIC = 1 mg/L). The transfer frequency was calculated as the number of transconjugant/number of recipient bacteria. Transconjugants were verified by PCR with specific primers: *bla*_NDM-1_-F(5’-GGTTTGGCGATCTGGTTTTC-3’), *bla*_NDM-1_-R (5’- CGGAATGGCTCATCACGATC-3’), *mcr-9*-F (5’- ATGCCTGTACTTTTCAGG-3’), and *mcr-9*-R (5’- CGATTAGCCACGGCATTC-3’).

### S1-PFGE and Southern blot

S1-PFGE was employed to characterize the plasmid profiles of the three isolates^35^. To determine the genomic localization, plasmid types, and sizes of the *bla*_NDM-1_ and *mcr-9* genes, S1-PFGE followed by Southern blot hybridization was performed. Briefly, total DNA was embedded in 1% SeaKem® Gold Agarose (Lonza, USA) and digested with S1 nuclease (Takara, Japan) at 37°C for 30 min to linearize supercoiled plasmids. The digested DNA was separated using a CHEF Mapper system (Bio-Rad, USA) for 18 h, with *Salmonella enterica* serotype Braenderup strain H9812 (*Xba*I-digested) serving as the molecular weight standard. Following electrophoresis, DNA fragments were transferred onto a positively charged nylon membrane (Roche, Germany) via capillary transfer. Hybridization was carried out using digoxigenin-labeled specific probes targeting *bla*_NDM-1_ and *mcr-9*. Probe labeling, hybridization, and signal detection were performed according to the instructions of the DIG-High Prime DNA Labeling and Detection Starter Kit I (Roche, Germany), enabling the localization of the target resistance genes.

### Whole-genome sequencing, database screening, and bioinformatic analysis

Genomic DNA was extracted from selected strains using a commercial DNA extraction kit (Generay, Shanghai, China). The extracted DNA was subjected to whole-genome sequencing using both Illumina and Oxford Nanopore GridION platforms and subsequently assembled by Unicycler v0.5.0 (Supplementary Data 2). In addition, Easyfig 2.2.5 (minimum e-value of 0.001, percent homology threshold = 99%) and BRIG 0.95 (homology threshold = 70%) were used for comparative plasmid analysis.

We screened 8,037 *E. hormaechei* genomes from NCBI (accessed September 30, 2024). Using ABRicate (https://github.com/tseemann/abricate), with the ResFinder database (default settings), we identified 1574 *bla*_NDM_-harboring isolates (carbapenem resistance) and 1531 *mcr*-carrying isolates, for which we systematically collected metadata including isolation country, year, source, MLST, and accession numbers using Python scripts and compiled into a comprehensive dataset (Supplementary Data 3).

All isolates (both experimental and database-derived) were identified for plasmid type as well as virulence factors (VFs) using ABRicate (https://github.com/tseemann/abricate). MLST of the isolates was determined using MLST v.2.30 (https://github.com/tseemann/mlst). The minimum spanning tree based on the allelic profiles of MLST was constructed using PHYLOViZ v.2.0.

### Phylogenetic analysis

To resolve the evolutionary relationships among the ST171, ST78, and ST231 lineages, a core-genome SNP-based phylogenetic analysis was performed. Genomic reads were mapped against their respective reference genomes—BC37, Eh1 (GCA_009834325.1), and BC18—using Snippy v4.3.6 with default parameters. The resulting core SNP alignment was used to infer a maximum-likelihood phylogeny with IQ-TREE^36^ v1.6.12. ModelFinder was first applied to select the best-fit substitution model, and branch support was determined via 1,000 ultrafast bootstrap replicates. To further delineate population structure, we applied hierarchical Bayesian clustering with RhierBAPS v1.0.1^37,38^, which partitioned the strain collection into genetically distinct subgroups based on allelic profiles. Finally, pairwise SNP distances between strains were subsequently calculated using snp-dists v.0.7.0 (https://github.com/tseemann/snp-dists) **(**Supplementary Data 7, 8**)**.

### Statistics and reproducibility

Statistical analysis was performed in R v.4.4.3. Spearman’s correlation analysis was used to determine the two-by-two correlation between ARGs, virulence genes and plasmid replicons. Sample sizes for each analysis are specified in the corresponding figure legends.

Conjugation assays were performed in three independent biological replicates, and transfer frequencies are reported as mean ± standard deviation (SD). S1-PFGE and Southern blot experiments were performed once, and representative images are shown.

### Reporting summary

Further information on research design is available in the Nature Portfolio Reporting Summary linked to this article.

## Supplementary information

Supplementary Information

Description of Additional Supplementary Files

Supplementary Data 1

Supplementary Data 2

Supplementary data 3

Supplementary Data 4

Supplementary Data 5

Supplementary Data 6

Supplementary data 7

Supplementary data 8

Reporting Summary

## Data Availability

The genome sequences of strains BC18, BC30 and BC37 were deposited at GenBank under accession numbers CP189976-CP189981, JBNLZP000000000, and CP189885-CP189887, respectively. Publicly available genome sequences used in this analysis are listed with their accession numbers in Supplementary Data 3. Source data underlying Figs. 3b–e, g–j, and 4–5 are provided in Supplementary Data 3. Source data underlying Fig. 6b, c are presented in Supplementary Data 7 and 8. All other data supporting the findings of this study are available from the corresponding author upon reasonable request. Uncropped images for the blots/gels are provided in Supplementary Fig. 4.
