# Supplementary material for: Global genomic and molecular epidemiology of carbapenem-resistant Enterobacter hormaechei
Source: Commun Biol. 2026 Apr 15;9:1071. doi: 10.1038/s42003-026-09895-2 (PMC13458537; doi:10.1038/s42003-026-09895-2)
Supplement: Supplementary file 1 [file 42003_2026_9895_MOESM11_ESM.pdf]

nature portfolio

Corresponding author(s): Biao Tang

Last updated by author(s): Jan 5, 2026

Reporting Summary

Nature Portfolio wishes to improve the reproducibility of the work that we publish. This form provides structure for consistency and transparency in reporting. For further information on Nature Portfolio policies, see our Editorial Policies and the Editorial Policy Checklist.

Statistics

For all statistical analyses, confirm that the following items are present in the figure legend, table legend, main text, or Methods section.

n/a	Confirmed	
☐	☒	The exact sample size (*n*) for each experimental group/condition, given as a discrete number and unit of measurement
☒	☐	A statement on whether measurements were taken from distinct samples or whether the same sample was measured repeatedly
☐	☒	The statistical test(s) used AND whether they are one- or two-sided<br>*Only common tests should be described solely by name; describe more complex techniques in the Methods section.*
☒	☐	A description of all covariates tested
☒	☐	A description of any assumptions or corrections, such as tests of normality and adjustment for multiple comparisons
☐	☒	A full description of the statistical parameters including central tendency (e.g. means) or other basic estimates (e.g. regression coefficient) AND variation (e.g. standard deviation) or associated estimates of uncertainty (e.g. confidence intervals)
☐	☒	For null hypothesis testing, the test statistic (e.g. *F*, *t*, *r*) with confidence intervals, effect sizes, degrees of freedom and *P* value noted<br>*Give P values as exact values whenever suitable.*
☒	☐	For Bayesian analysis, information on the choice of priors and Markov chain Monte Carlo settings
☒	☐	For hierarchical and complex designs, identification of the appropriate level for tests and full reporting of outcomes
☐	☒	Estimates of effect sizes (e.g. Cohen's *d*, Pearson's *r*), indicating how they were calculated

Our web collection on statistics for biologists contains articles on many of the points above.

Software and code

Policy information about availability of computer code

Data collection	8, 037 publicly available E. hormaechei genomes were downloaded from the NCBI database (accessed September 30, 2024).
Data analysis	The extracted DNA was subjected to whole-genome sequencing using both Illumina and Oxford Nanopore GridION platforms and subsequently assembled by Unicycler v0.5.0. In addition, Easyfig 2.2.5 (minimum e-value of 0.001, percent homology threshold = 99%) and BRIG 0.95 (homology threshold = 70%) were used for comparative plasmid analysis. All isolates (both experimental and database-derived) isolates were identified for plasmid type as well as virulence factors (VFs) using ABRicate (https://github.com/tseemann/abricate). The multilocus sequence type (MLST) of the isolates was determined using MLST v.2.30 (https://github.com/tseemann/mlst). The minimum spanning tree based on the allelic profiles of MLST were constructed using PHYLOViZ v.2.0. The genomic sequences of the strains classified as ST171, ST78 and ST231 were compared with the reference genomes BC37, Eh1(GCA 009834325) and BC18 using Snippy v4.3.6 with default parameters. A maximum-likelihood (ML) phylogenetic tree was inferred using IQ-TREE v1.6.12 under the best-fit substitution model (selected by ModelFinder) with 1, 000 bootstrap replicates to assess branch support. Population structure was further analyzed through hierarchical Bayesian clustering implemented in RhierBAPS v1.0.1, which partitions strains into genetically distinct subgroups based on allelic variation. SNPs were detected in the strain genome using snp-dists v.0.7.0.

For manuscripts utilizing custom algorithms or software that are central to the research but not yet described in published literature, software must be made available to editors and reviewers. We strongly encourage code deposition in a community repository (e.g. GitHub). See the Nature Portfolio guidelines for submitting code & software for further information.

Data

Policy information about availability of data

All manuscripts must include a data availability statement. This statement should provide the following information, where applicable:

- Accession codes, unique identifiers, or web links for publicly available datasets
- A description of any restrictions on data availability
- For clinical datasets or third party data, please ensure that the statement adheres to our policy

The complete genome sequences of strains BC18, BC30 and BC37 were deposited at GenBank under accession numbers CP189976-CP189981, JBNLZP000000000, CP189885- CP189887, respectively.

Research involving human participants, their data, or biological material

Policy information about studies with human participants or human data. See also policy information about sex, gender (identity/presentation), and sexual orientation and race, ethnicity and racism.

Reporting on sex and gender	Not Applicable
Reporting on race, ethnicity, or other socially relevant groupings	Not Applicable
Population characteristics	Not Applicable
Recruitment	Not Applicable
Ethics oversight	All activities conducted in this study received approval from the Research Ethics Committee of Zhengzhou People's Hospital (KY2022- 013-01).

Note that full information on the approval of the study protocol must also be provided in the manuscript.

Field-specific reporting

Please select the one below that is the best fit for your research. If you are not sure, read the appropriate sections before making your selection.

☒ Life sciences ☐ Behavioural & social sciences ☐ Ecological, evolutionary & environmental sciences

For a reference copy of the document with all sections, see nature.com/documents/nr-reporting-summary-flat.pdf

Life sciences study design

All studies must disclose on these points even when the disclosure is negative.

Sample size	No formal sample size calculation was conducted. The sample size was determined based on the number of bacterial isolates obtained from clinical specimens submitted to the clinical microbiology laboratory of a tertiary hospital located in Henan Province, China. The number of genomes included in the analysis was determined by the availability of high-quality sequencing data.
Data exclusions	No data were excluded from the analyses. All isolates collected and genomes sequenced were included.
Replication	Replication is not relevant because all genomes that were sequenced were included in the analyses.
Randomization	Randomization is not relevant because all genomes that were sequenced were included in the analyses.
Blinding	Randomization is not relevant because all genomes that were sequenced were included in the analyses.

Reporting for specific materials, systems and methods

We require information from authors about some types of materials, experimental systems and methods used in many studies. Here, indicate whether each material, system or method listed is relevant to your study. If you are not sure if a list item applies to your research, read the appropriate section before selecting a response.

Materials & experimental systems

n/a	Involved in the study
☒	☐ Antibodies
☒	☐ Eukaryotic cell lines
☒	☐ Palaeontology and archaeology
☒	☐ Animals and other organisms
☒	☐ Clinical data
☒	☐ Dual use research of concern
☒	☐ Plants

Methods

n/a	Involved in the study
☒	☐ ChIP-seq
☒	☐ Flow cytometry
☒	☐ MRI-based neuroimaging

Plants

Seed stocks	Not applicable
Novel plant genotypes	Not applicable
Authentication	Not applicable
