# Supplementary material for: Global genomic and molecular epidemiology of carbapenem-resistant Enterobacter hormaechei
Source: Commun Biol. 2026 Apr 15;9:1071. doi: 10.1038/s42003-026-09895-2 (PMC13458537; doi:10.1038/s42003-026-09895-2)
Supplement: Supplementary file 2 [file 42003_2026_9895_MOESM1_ESM.pdf]

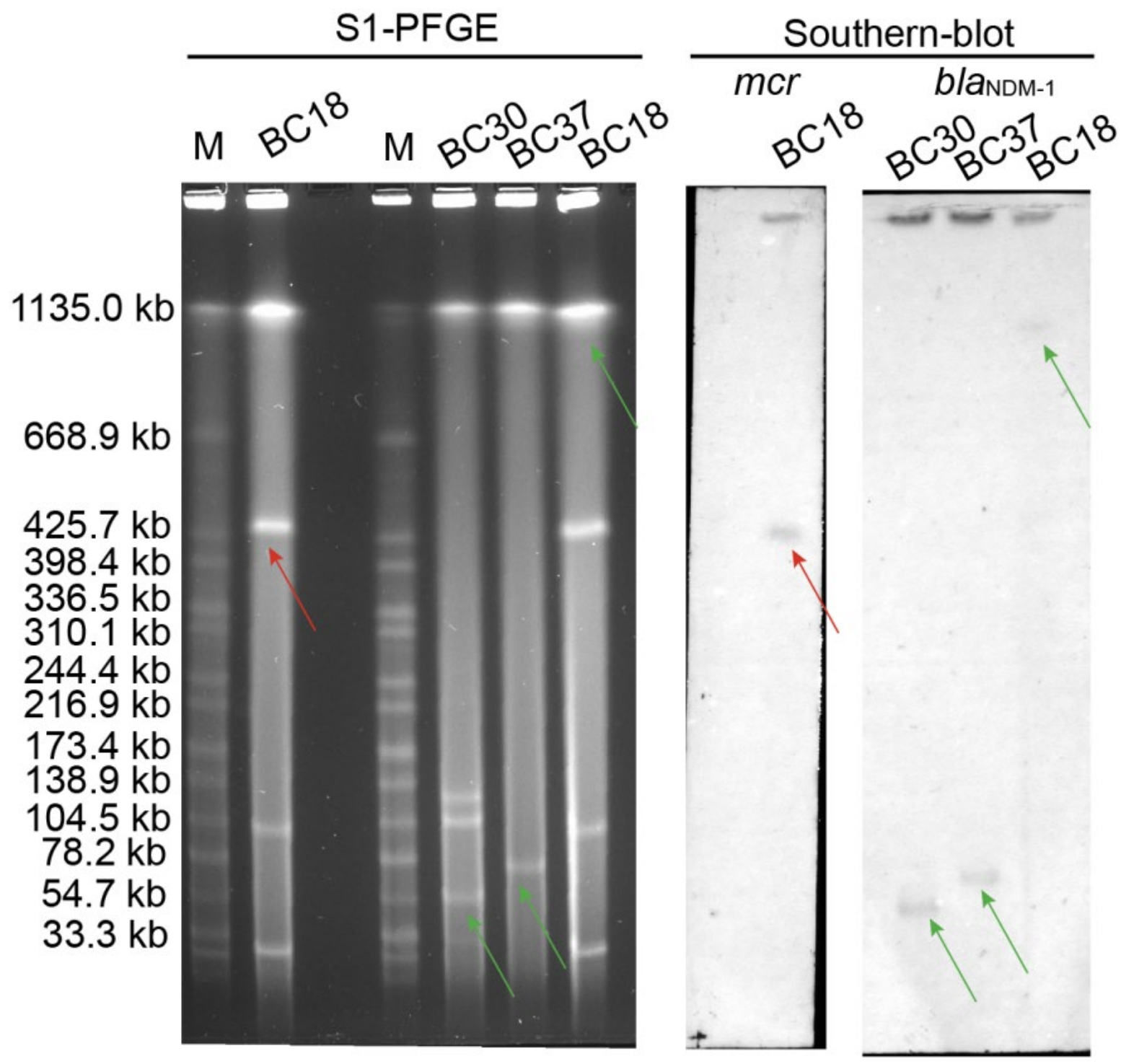

Supplementary Figure 1. Plasmid size and confirmation of *mcr-1* and bla_{NDM-1} genes. **(A)** S1-PFGE of strains BC18, BC30 and BC37. **(B)** Southern blot of strains BC18, BC30 and BC37. The plasmid harboring bla_{NDM-1} gene was indicated with red arrows. The plasmid harboring *mcr* gene was indicated with green arrows.

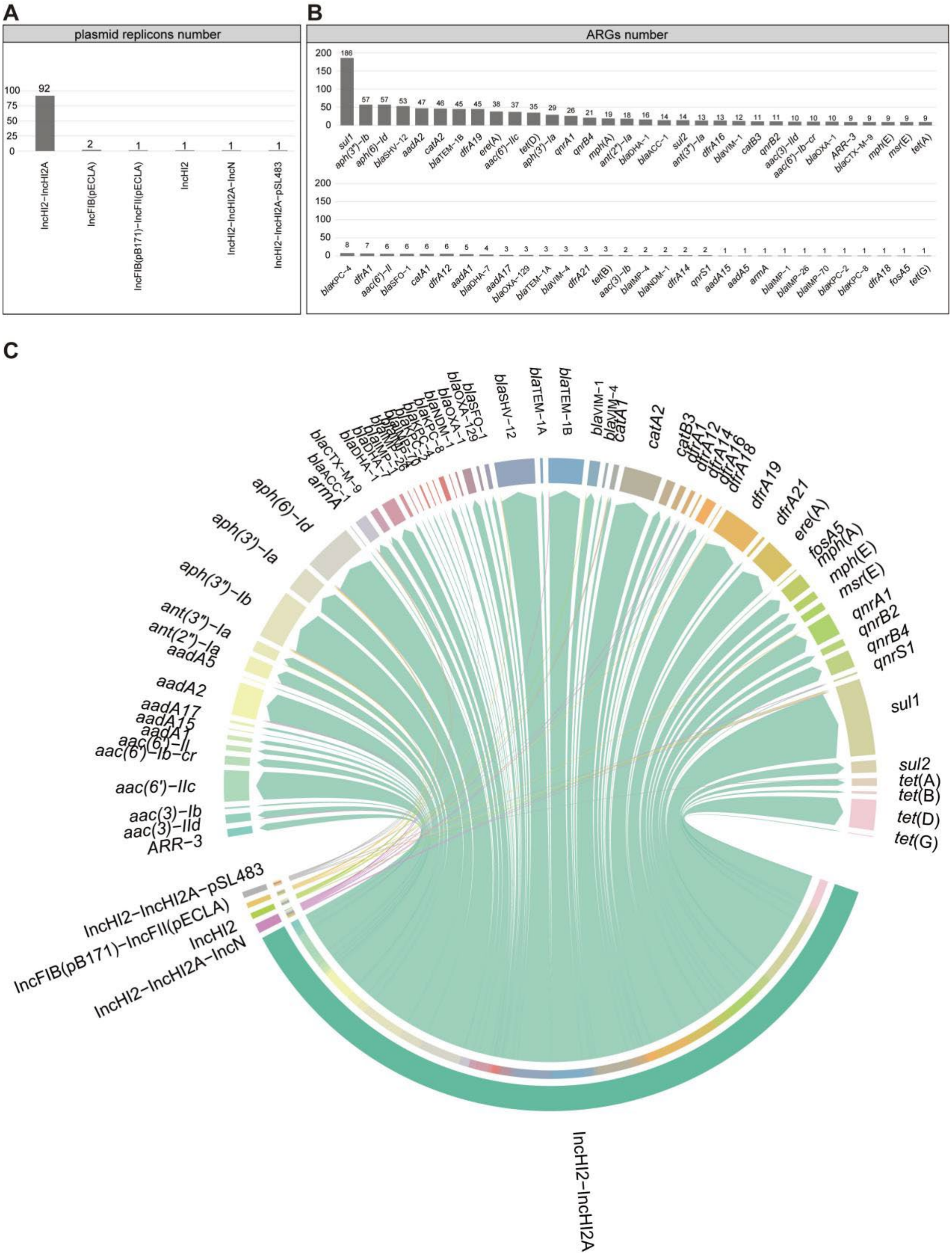

Supplementary Figure 2. Distribution and co-occurrence of ARGs and plasmid replicons of 98 contigs carrying *mcr-9* gene. **(A)** Distribution of plasmid replicons among *mcr-9*–positive contigs (n=98). **(B)** Distribution of plasmid-borne ARG numbers in *mcr-9*–positive contigs (n=98). **(C)** String plot of co-occurrence of ARGs and plasmid replicons.

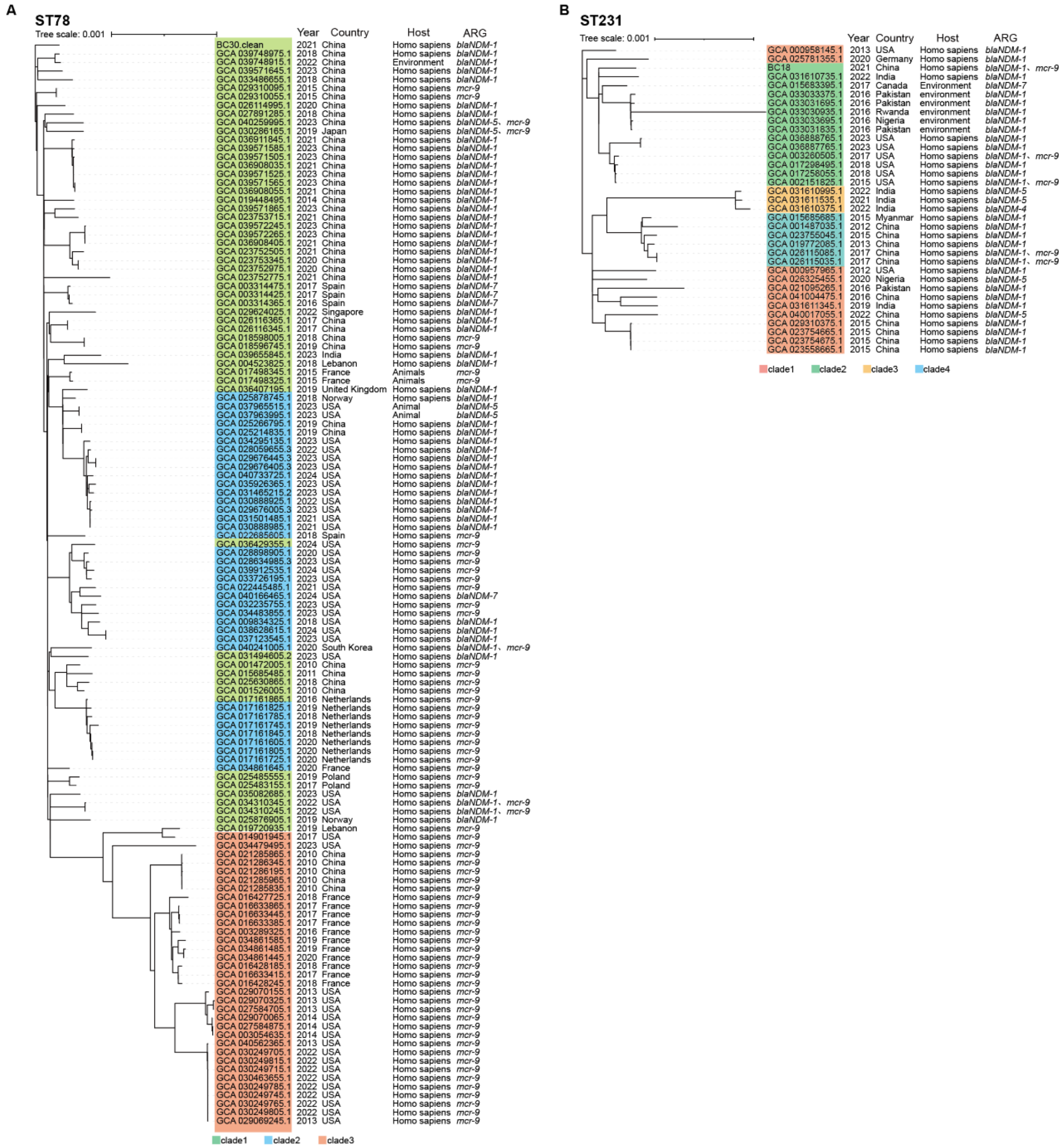

Supplementary Figure 3. Phylogenetic tree of ST78 and ST231 isolates. **(A)** Phylogenetic tree of 126 isolates belonging to ST78 subtype. **(B)** Phylogenetic tree of 35 isolates belonging to ST231 subtype. Different branch colors represent different clade. The information shown in the figure is as follows: the accession number of the strain, year, country, source, and the situation where the strain carries the *bla*NDM and *mcr* genes. Different rectangular colors in the developmental tree represent different clusters.

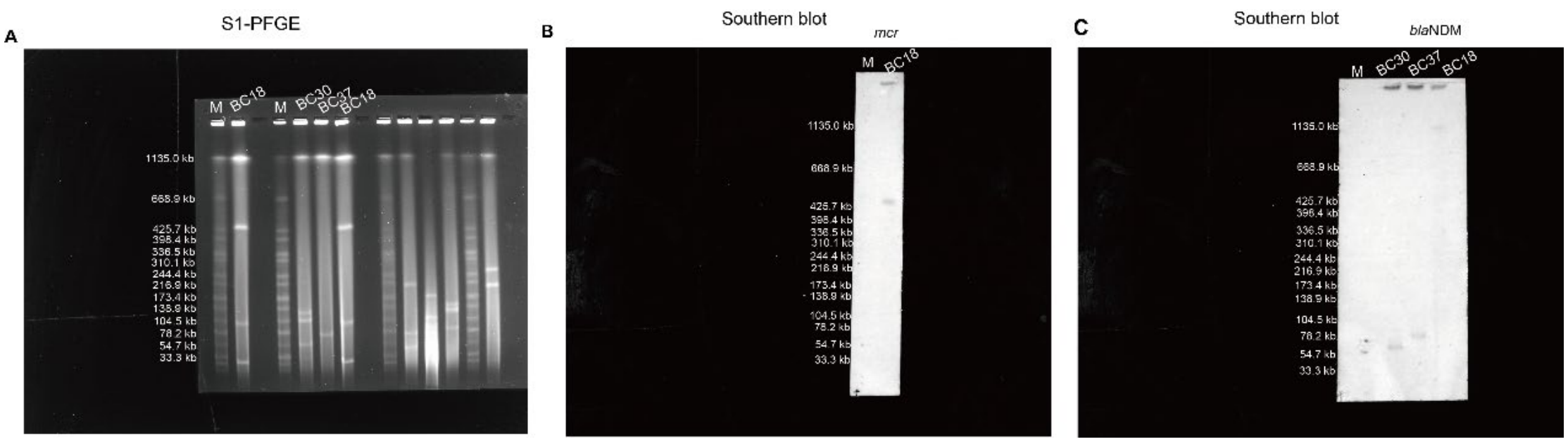

Supplementary Figure 4. Original and uncropped images corresponding to Supplementary Figure 1. **(A)** Full S1-PFGE gel image. **(B–C)** Original southern blot images for **(B)** *mcr-9* and **(C)** bla_{NDM-1}. In all panels, molecular weight markers are indicated on the left.
