# Supplementary material for: Global genomic and molecular epidemiology of carbapenem-resistant Enterobacter hormaechei
Source: Commun Biol. 2026 Apr 15;9:1071. doi: 10.1038/s42003-026-09895-2 (PMC13458537; doi:10.1038/s42003-026-09895-2)
Supplement: Supplementary file 3 [file 42003_2026_9895_MOESM2_ESM.pdf]

Description of Additional Supplementary Files:

File: Supplementary Data 1

Description: Phenotypic antimicrobial susceptibility profiles of the three E. hormaechei isolates.

File: Supplementary Data 2

Description: Comparative genomic characterization of the three study isolates.

File: Supplementary Data 3

Description: Metadata and genomic characteristics of blaNDM- and mcr-haboring E. hormaechei

File: Supplementary Data 4

Description: Accession numbers and replicons of plasmids carrying the blaNDM gene.

File: Supplementary Data 5

Description: Genomic context and characteristics of the mcr-9-carrying plasmids.

File: Supplementary Data 6

Description: Frequency of cooccurrence between plasmid replicons and additional ARGs in mcr-9-carrying isolates.

File: Supplementary Data 7

Description: Transmission matrix of strains with SNPs < 5.

File: Supplementary Data 8

Description: Transmission matrix of strains with 5 ≤ SNPs < 20.
